# Assessing Climate Change Impacts on the Distribution and Ecological Risks of Cryptophyta in the China Seas Using Ensemble Models

**DOI:** 10.3390/biology15131047

**Published:** 2026-07-01

**Authors:** Ru Lan, Jing Li, Rongchang Chen, Zhentian Cai, Luning Li

**Affiliations:** 1 Environmental Protection & Energy-Saving Technique Research Center, China Waterborne Transport Research Institute, Beijing 100088, China; lanru@wti.ac.cn (R.L.); chenrongchang@wti.ac.cn (R.C.); 2 School of Energy and Environmental Engineering, University of Science and Technology Beijing, Beijing 100083, China; 3 Ocean and Food School, Quanzhou Normal University, Quanzhou 362000, China; leekin@qztc.edu.cn; 4 Key Laboratory of Coastal Resource Biotechnology, Fujian Higher Institution, Quanzhou 362000, China; 5 Jinjiang College, Sichuan University, Meishan 620860, China; 18990186869@163.com

**Keywords:** Cryptophyta, habitat suitability, Biomod2, ballast water, environmental drivers, coastal surveillance

## Abstract

Cryptophyta are ecologically important microalgae in marine and brackish ecosystems, but their future habitat suitability and ballast-water-associated surveillance priorities under climate change remain insufficiently understood. Based on 136 georeferenced occurrence records from China’s coastal waters and 24 marine environmental variables, this study applied the Biomod2 ensemble modeling framework to predict the current and future potential distribution of Cryptophyta in the Bohai Sea, Yellow Sea, East China Sea, and South China Sea. The ensemble model showed high predictive performance (Kappa = 0.94, TSS = 0.94, AUC = 0.997), outperforming most individual algorithms. Annual mean temperature (bio22) and maximum salinity (bio15) were identified as the dominant environmental constraints, indicating that thermal and salinity regimes jointly shape habitat suitability. Under present conditions, suitable habitats were broadly distributed across China’s coastal and shelf waters. Future projections indicated that suitable habitats generally remained extensive under most climate scenarios, suggesting that future environmental changes may maintain or reshape potential recipient environments for Cryptophyta. However, this response was not linear with increasing emission intensity; under the high-emission scenario, suitable habitats declined in some regions and showed stronger spatial reorganization, implying that excessive warming or altered salinity may reduce broad-scale suitability. These findings suggest that climate change may alter the spatial pattern of Cryptophyta habitat suitability under different emission pathways. Given the role of ship ballast water in transporting planktonic microalgae, persistent or newly suitable habitats, especially those located near ports, estuaries, semi-enclosed bays, and aquaculture areas, should be regarded as habitat-suitability-based surveillance priorities. This study provides a basis for climate-informed monitoring, ballast-water-associated screening, and adaptive management of coastal microalgae in the China Seas.

## Simple Summary

Cryptophytes are common microscopic algae in coastal waters and important components of marine food webs, but changes in their distribution may affect plankton communities and coastal ecosystem stability. This study used an ensemble species distribution model to predict the current and future potential distribution of Cryptophyta in the China Seas. Annual mean temperature and maximum salinity were the main factors shaping habitat suitability. Suitable habitats are projected to remain extensive and increase slightly under low- to moderate-emission scenarios, from the current 63.55 × 10^4^ km^2^ to approximately 66 × 10^4^ km^2^, whereas the high-emission scenario by the 2100s shows habitat contraction and stronger spatial reorganization. These results suggest a scenario-dependent response to climate change rather than simple expansion. Because ship ballast water can transport microalgae over long distances, persistent or newly suitable habitats overlapping with ports, estuaries, semi-enclosed bays, and aquaculture areas should be prioritized for long-term monitoring.

## 1. Introduction

Several microalgal groups, including diatoms, dinoflagellates, cyanobacteria, chlorophytes, haptophytes, and cryptophytes, are widely distributed in marine and coastal ecosystems. Cryptophytes are eukaryotic unicellular flagellated microalgae belonging to the class Cryptophyceae within Cryptista. They are widely distributed in marine, brackish, and freshwater ecosystems, where they play important roles in primary production, nutrient cycling, and planktonic food-web energy transfer [1,2]. Although traits such as small cell size, rapid population response, and tolerance to environmental fluctuation are not unique to Cryptophyta, this group is relevant to ballast-water-associated risk assessment because it has been recorded among suspected alien microalgae requiring attention in Chinese coastal ports [3,4,5]. Previous ballast-tank simulation observations further suggest that cryptophyte cells can persist under low-temperature and dark conditions and recover growth after exposure to suitable receiving-water environments. Ship ballast water and ballast-tank sediments can transport viable algal cells across biogeographic barriers, thereby increasing propagule pressure in receiving coastal waters [6,7]. Once discharged into estuaries, semi-enclosed bays, aquaculture zones, or shelf waters with suitable environmental conditions, transported Cryptophyta may survive or reinforce local populations, although establishment depends on propagule supply, species identity, biological interactions, and local environmental conditions [8]. Therefore, Cryptophyta represents an ecologically important phytoplankton group and a relevant taxonomic group for assessing recipient-habitat suitability associated with ballast-water-mediated microalgal dispersal.

Climate change is expected to further modify the environmental conditions that determine the survival and establishment potential of Cryptophyta. Global mean sea surface temperature has already increased by approximately 0.88 °C from pre-industrial levels (1850–1900) to the present decade, and recent climate projections indicate continued ocean warming under low- to high-emission pathways by the end of the 21st century [9]. Ocean warming, salinity redistribution, changes in coastal circulation, and altered water-column stability may reshape the suitability of coastal habitats for phytoplankton [10]. For Cryptophyta, whose distribution is closely linked to thermal and salinity gradients, future climate change may expand suitable habitats in some regions, reduce suitability in others, or shift habitat centers toward newly favorable coastal waters [10]. Such changes are particularly important in the China Seas, where dense port networks, intensive international shipping, major estuaries, aquaculture activities, and semi-enclosed coastal systems coincide with strong environmental gradients [11]. In this context, evaluating only present-day occurrence patterns is insufficient. Predicting future habitat suitability is necessary to identify where climate-driven environmental changes may create recipient environments in which ballast-water introductions would require closer surveillance.

Species distribution models (SDMs) provide an effective approach for linking species occurrence records with environmental variables and projecting potential distributions under current and future climate conditions [12]. However, predictions based on a single algorithm may be affected by model assumptions, sampling bias, and algorithm-specific uncertainty [13]. Ensemble modeling integrates multiple algorithms and can improve predictive robustness by reducing dependence on any single model structure [14,15]. This approach is especially suitable for broad-scale assessments of microalgal taxa, for which occurrence records are often spatially uneven and responses to environmental gradients may be complex [16]. By combining multiple modeling algorithms, ensemble SDMs can provide a more stable basis for identifying suitable habitats, environmental drivers related to temperature, salinity, hydrodynamics, and low-temperature seasonal constraints, and future distribution shifts.

Despite the ecological importance and potential ballast-water relevance of Cryptophyta, large-scale assessments of its current and future habitat suitability in the China Seas remain limited [16,17]. In particular, it is still unclear where suitable habitats are currently concentrated, which environmental variables most strongly constrain their distribution, how suitable areas may change under future climate scenarios, and which coastal regions may require priority monitoring. Therefore, the main objective of this study was to assess the current and future potential habitat suitability of Cryptophyta in the China Seas using an ensemble species distribution modeling framework, with particular attention to ballast-water-associated surveillance priorities. Specifically, we aimed to: (1) predict the current potential habitat suitability of Cryptophyta in the China Seas; (2) identify the key environmental variables shaping its distribution; (3) project changes in suitable habitats under future climate scenarios for the 2050s and 2100s; and (4) evaluate habitat expansion, contraction, persistence, and centroid migration. By integrating climate-change projections with a ballast-water-associated monitoring perspective, this study provides a scientific basis for early warning, port-focused surveillance, and long-term management of potential recipient habitats in China’s coastal waters.

## 2. Materials and Methods

### 2.1. Study Area and Cryptophyta Occurrence Records

This study focused on the four major marginal seas of China, including the Bohai Sea, Yellow Sea, East China Sea, and South China Sea. These coastal waters are environmentally heterogeneous, covering latitudinally and hydrographically distinct marginal seas with different temperatures, salinities, hydrodynamics, and seasonal conditions. They also include dense port networks, major estuaries, aquaculture areas, and intensive international shipping routes. Because these regions are strongly influenced by port activities and ballast-water discharge [11,18], they provide a representative setting for assessing the potential habitat suitability and surveillance relevance of ballast-water-associated Cryptophyta under current and future climate conditions.

Cryptophyta occurrence records were obtained from a database of suspected alien microalgae in Chinese coastal port waters. The database integrated field investigations conducted during 2017–2022 and environmental impact assessment reports and related coastal monitoring records collected during 2012–2022. These records included observations from coastal phytoplankton surveys, port-water monitoring, ballast-water-related investigations, and ballast-tank sediment samples. Because many phytoplankton datasets identify cryptophytes only at broad taxonomic levels, Cryptophyta was modeled as a taxonomic group rather than as individual species. The resulting predictions therefore describe broad habitat suitability patterns for Cryptophyta across the China Seas and provide a group-level screening of environmentally suitable recipient areas. This group-level treatment may integrate ecological signals from cryptophyte taxa with different environmental tolerances, and species-level niche differentiation should be further resolved using finer taxonomic identification and standardized occurrence records.

All occurrence records were checked for taxonomic consistency, coordinate accuracy, and spatial validity. Records with missing coordinates, duplicated coordinates, ambiguous geographic information, vague locality descriptions, or locations outside the marine study area were excluded. After quality control, 136 valid georeferenced occurrence records were retained for model construction. No additional spatial thinning was applied beyond the removal of duplicated coordinates and invalid geographic records. The retained occurrence records, including longitude and latitude, are provided in Supplementary Table S3. All occurrence records were standardized to the WGS84 geographic coordinate system and matched with environmental raster layers before modeling.

### 2.2. Environmental Variables and Climate Scenarios

A total of 24 marine environmental variables were initially collected to represent the main hydrographic and climatic gradients potentially affecting the survival, establishment, and distribution of Cryptophyta (Table 1). These variables covered four categories: current velocity, ice thickness, salinity, and temperature. Current-related variables were used to describe hydrodynamic conditions and potential dispersal processes; ice-related variables reflected seasonal low-temperature constraints in northern coastal waters; salinity variables represented osmotic and estuarine gradients; and temperature variables described thermal conditions associated with algal growth and habitat suitability.

Present-day environmental layers were obtained from Bio-ORACLE v2.1 and the World Ocean Atlas 2018 (WOA2018), which provide long-term climatological marine environmental data for bioclimatic and oceanographic analyses [19,20]. Bio-ORACLE v2.1 provides global marine environmental raster layers for surface and benthic realms at a spatial resolution of 5 arcmin, whereas WOA2018 provides climatological oceanographic products, including temperature and salinity, at standard depth levels and different grid resolutions [19,20]. Because the occurrence records used in this study were mainly derived from coastal phytoplankton and ballast-water-associated surveys, surface marine layers were selected to represent the environmental conditions most relevant to Cryptophyta occurrence in coastal and shelf waters. Future environmental layers were selected under three Representative Concentration Pathway scenarios, namely RCP2.6, RCP4.5, and RCP8.5, representing low-, medium-, and high-emission pathways, respectively [21]. Future layers for the 2050s and 2100s were obtained from the processed Bio-ORACLE climate products; therefore, no additional general circulation model selection, bias correction, or statistical downscaling was performed in this study. To improve reproducibility, detailed information on the six retained predictors used in the final ensemble model, including variable name, unit, layer setting, original data source, original resolution, processed resolution, temporal baseline, and future scenario source, is provided in Table S4.

All environmental raster layers were clipped to the China Seas marine study area, standardized to the WGS84 geographic coordinate system, resampled to a common 5 arcmin grid, and aligned to the same spatial extent, grid origin, cell size, and raster format before model construction. Continuous environmental variables were resampled using bilinear interpolation. Environmental values corresponding to occurrence and pseudo-absence/background points were extracted using ArcGIS v10.4.1 and R v4.3.2.

**Figure 1:**
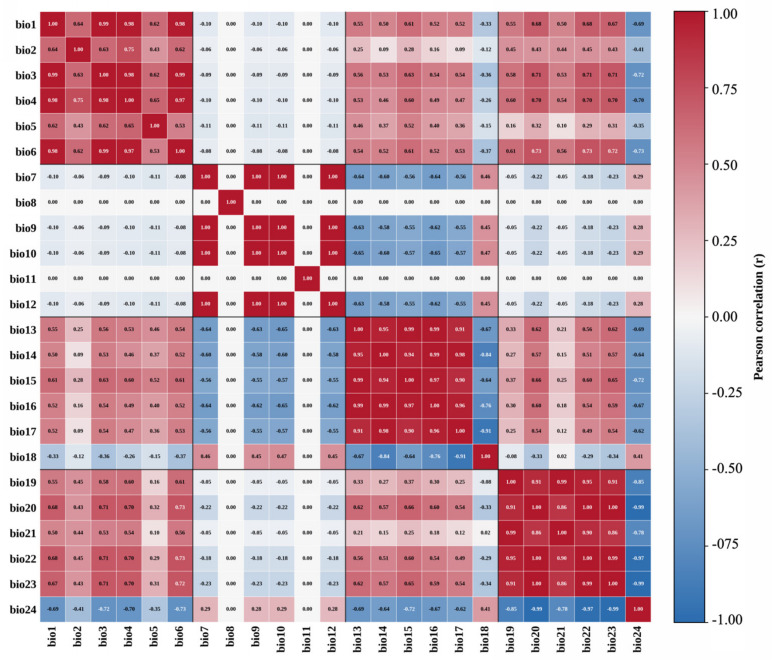
Pearson correlation heatmap of 24 candidate environmental variables. The color gradient indicates the correlation coefficient (r), with red representing positive correlations and blue representing negative correlations.

**Figure 2:**
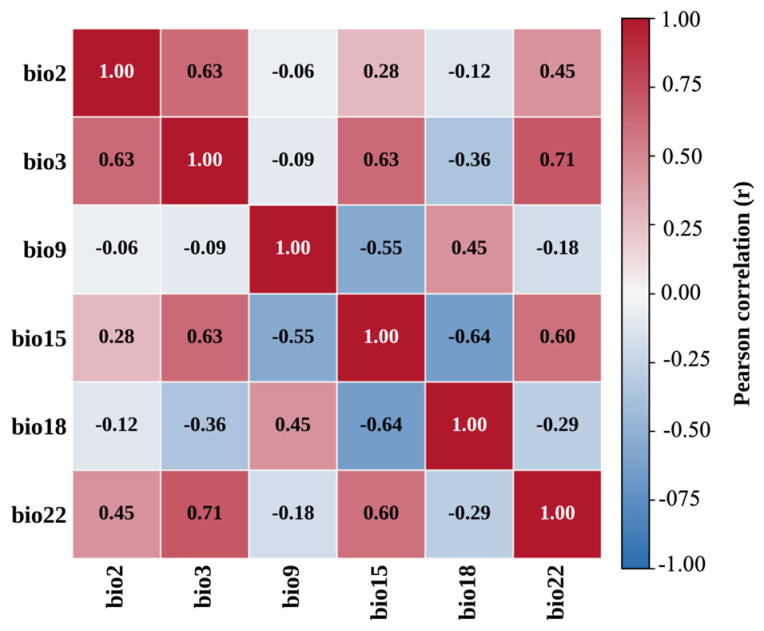
Pearson correlation heatmap of six key environmental variables retained after variable screening (bio2: minimum monthly mean current velocity; bio3: maximum current velocity; bio9: maximum ice thickness; bio15: maximum salinity; bio18: annual salinity range; bio22: annual mean temperature).

To reduce multicollinearity among environmental predictors, Pearson correlation analysis was conducted for the 24 candidate variables. The pairwise correlation structure among all candidate predictors is shown in Figure 1. When the absolute value of the correlation coefficient between two variables was greater than 0.8, the variables were considered highly correlated, and only one variable was retained based on ecological relevance, environmental interpretability, and its contribution to explaining Cryptophyta distribution. After screening, six variables were retained for final ensemble modeling: minimum monthly mean current velocity (bio2), maximum current velocity (bio3), maximum ice thickness (bio9), maximum salinity (bio15), annual salinity range (bio18), and annual mean temperature (bio22). The correlations among these six retained predictors are shown in Figure 2, and additional screening information for the excluded variables is provided in Supplementary Table S5. These variables jointly represent the hydrodynamic, cryospheric, salinity, and thermal constraints shaping the potential distribution of Cryptophyta in China’s coastal seas.

### 2.3. Ensemble Species Distribution Modeling

The potential distribution of Cryptophyta was modeled using the Biomod2 package in R v4.3.2. Compared with a single species distribution model, the ensemble modeling framework integrates multiple algorithms and reduces algorithm-specific uncertainty, thereby improving the robustness and reliability of habitat suitability prediction [15].

Nine commonly used species distribution modeling algorithms were included: generalized linear model (GLM), generalized boosted model (GBM), generalized additive model (GAM), classification tree analysis (CTA), artificial neural network (ANN), surface range envelope (SRE), multivariate adaptive regression splines (MARS), random forest (RF), and maximum entropy model (MaxEnt) [14]. MaxEnt was implemented through the MaxEnt.Phillips algorithm within the Biomod2 framework.

Because the Cryptophyta dataset consisted of presence-only occurrence records, true absence records were not available for model calibration. Therefore, pseudo-absence/background points were generated within the marine study area and used as contrast data together with the occurrence records. These points were used only to meet the input requirements of the Biomod2 ensemble algorithms and should not be interpreted as confirmed absence records. Given the heterogeneous sources of the historical occurrence records, the available metadata did not support the construction of a consistent sampling-effort layer for target-group background or bias-file correction. Detailed settings for pseudo-absence/background generation are provided in Supplementary Text S2. The modeling dataset was randomly divided into training and testing subsets, with 75% of the data used for model calibration and 25% used for model evaluation. To reduce uncertainty caused by random data partitioning, each individual model was repeated 10 times under the same modeling framework. This random-partition evaluation was used to assess internal model discrimination and was not intended to represent a fully spatially independent validation.

Model performance was evaluated using three indices: Cohen’s Kappa statistic, the true skill statistic (TSS), and AUC [22]. These indices were used to assess the discriminatory ability and predictive reliability of each individual model run. Only individual model runs with AUC ≥ 0.8 were retained for ensemble modeling; therefore, poorly performing runs, including SRE runs below this threshold, were excluded. The final ensemble prediction was generated using the AUC-weighted mean method, in which model runs with higher predictive performance contributed more strongly to the final habitat suitability output.

Variable importance was assessed using the permutation-based procedure implemented in Biomod2. For each predictor, values were randomly permuted, and the resulting changes in model prediction were compared with the original prediction. Higher importance values indicate a stronger contribution of the variable to Cryptophyta habitat suitability.

### 2.4. Habitat Suitability Classification and Area Statistics

The ensemble model generated continuous habitat suitability values ranging from 0 to 1, with higher values indicating greater suitability for Cryptophyta occurrence. Based on the maximum TSS criterion used for binary reclassification [23], areas with suitability values lower than 0.575 were classified as unsuitable habitats, whereas areas with suitability values equal to or greater than 0.575 were classified as suitable habitats. Suitable habitats were further divided into three classes: low-suitability habitats with suitability values of 0.575–0.6, moderate-suitability habitats with values of 0.6–0.8, and high-suitability habitats with values of 0.8–1.0.

For the present period and each future climate scenario, the areas of unsuitable, low-suitability, moderate-suitability, and high-suitability habitats were calculated using ArcGIS v10.4.1 after spatial projection and raster reclassification. To quantify temporal changes in habitat suitability, binary suitability maps for future scenarios were compared with the present-day suitability map. Areas that changed from suitable under present conditions to unsuitable under future scenarios were defined as contracted areas; areas that changed from unsuitable to suitable were defined as expanded areas; and areas that remained suitable in both periods were defined as unchanged suitable areas.

### 2.5. Centroid Migration Analysis

To evaluate spatial shifts in Cryptophyta suitable habitats under climate change, centroid migration analysis was performed using binary suitable habitat maps [24]. For the present period and each future climate scenario, suitable habitat patches were converted into spatial polygons, and the geographic centroid of the total suitable habitat was calculated. The centroid coordinates were then mapped in ArcGIS v10.4.1 to visualize the direction and magnitude of habitat movement.

The centroid coordinates were compared among the present period, 2050, and 2100 under RCP2.6, RCP4.5, and RCP8.5 scenarios. The direction and distance of centroid movement were used to characterize the spatiotemporal redistribution of Cryptophyta suitable habitats. This analysis provided additional spatial evidence for assessing whether future climate change may drive directional shifts in the potential distribution of Cryptophyta along China’s coastal waters.

## 3. Results

### 3.1. Performance of Individual Models and Ensemble Model

**Figure 3:**
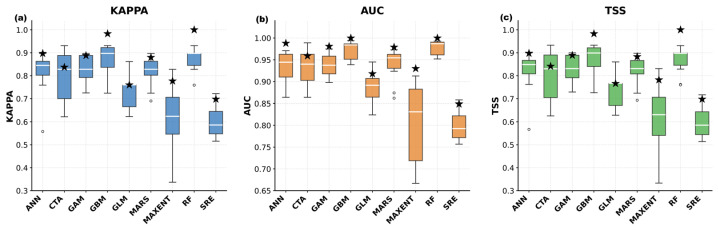
Comparison of prediction accuracy among different distribution models based on 10 replicated runs. (**a**) Kappa statistics, (**b**) area under the receiver operating characteristic curve (AUC), and (**c**) true skill statistic (TSS) were used to evaluate model performance. Boxplots show the variation among the 10 replicated runs, while black stars indicate the model evaluation values obtained using the full dataset.

The predictive performance of the nine individual algorithms and the ensemble model was evaluated using Kappa, TSS, and AUC based on ten replicated runs (Figure 3; Table 2). Overall, most algorithms showed good predictive ability for Cryptophyta distribution. RF and GBM performed best among the individual models, with mean AUC values of 0.978 and 0.973, respectively, and mean Kappa and TSS values above 0.86. GAM, MARS, ANN, and CTA also showed stable performance, with mean AUC values ranging from 0.931 to 0.938. GLM showed moderate accuracy, with a mean AUC of 0.885.

MaxEnt and SRE performed less consistently. MaxEnt had a mean AUC of 0.808 but showed relatively large variation among runs, whereas SRE had the lowest overall accuracy, with mean Kappa, TSS, and AUC values of 0.595, 0.594, and 0.797, respectively. These results indicate that model performance varied among algorithms, supporting the use of an ensemble approach.

The ensemble model (EMwmean), constructed from qualified models with AUC values of 0.8 or higher, achieved high predictive accuracy, with Kappa = 0.94, TSS = 0.94, and AUC = 0.997. Therefore, the EMwmean prediction was used for subsequent analyses of environmental drivers, habitat suitability, future distribution changes, habitat dynamics, and centroid migration.

### 3.2. Environmental Drivers of Cryptophyta Habitat Suitability

**Figure 4:**
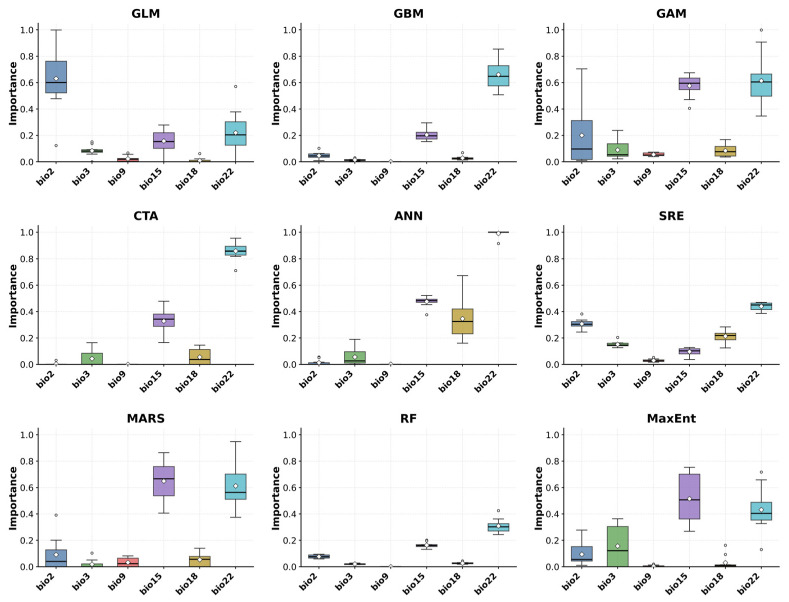
Distribution of variable importance for six key environmental predictors (bio2, bio3, bio9, bio15, bio18, bio22) across nine algorithms.

**Figure 5:**
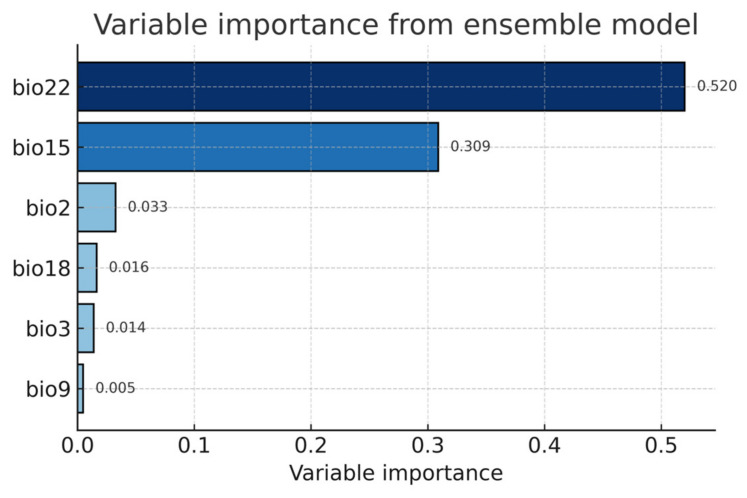
Relative importance of six key environmental factors in the ensemble model.

Variable-importance analysis showed that temperature and salinity were the dominant environmental drivers of Cryptophyta distribution (Figure 4 and Figure 5). Among the six retained predictors, annual mean temperature (bio22) had the highest importance in the ensemble model, with an importance value of 0.520 (52.0%), followed by maximum salinity (bio15), with an importance value of 0.309 (30.9%). This result indicates that broad-scale thermal conditions and salinity structure are the principal environmental constraints shaping Cryptophyta habitat suitability in the China Seas. Because Cryptophyta are sensitive to changes in water temperature and osmotic conditions, these two variables likely determine both the spatial extent of suitable habitats and the stability of nearshore populations [25].

The remaining predictors had much lower importance values. Minimum monthly mean current velocity (bio2) contributed 0.033 (3.3%), followed by annual salinity range (bio18; 0.016, 1.6%), maximum current velocity (bio3; 0.014, 1.4%), and maximum ice thickness (bio9; 0.005, 0.5%). Although their contributions were limited, current-related variables may still influence local dispersal, retention, and accumulation of cryptophyte cells in semi-enclosed coastal waters [26]. The low contribution of ice thickness suggests that ice-related constraints were not the primary factor controlling the overall distribution pattern within the study area.

Overall, the single-model and ensemble-model results consistently identified annual mean temperature and maximum salinity as the key variables shaping the potential distribution of Cryptophyta. These findings suggest that future changes in warming intensity, coastal salinity gradients, and water-mass exchange may strongly affect the redistribution of suitable habitats, providing an environmental basis for interpreting the current distribution pattern and projected changes under climate scenarios.

### 3.3. Current Potential Habitat Suitability of Cryptophyta in the China Seas

**Figure 6:**
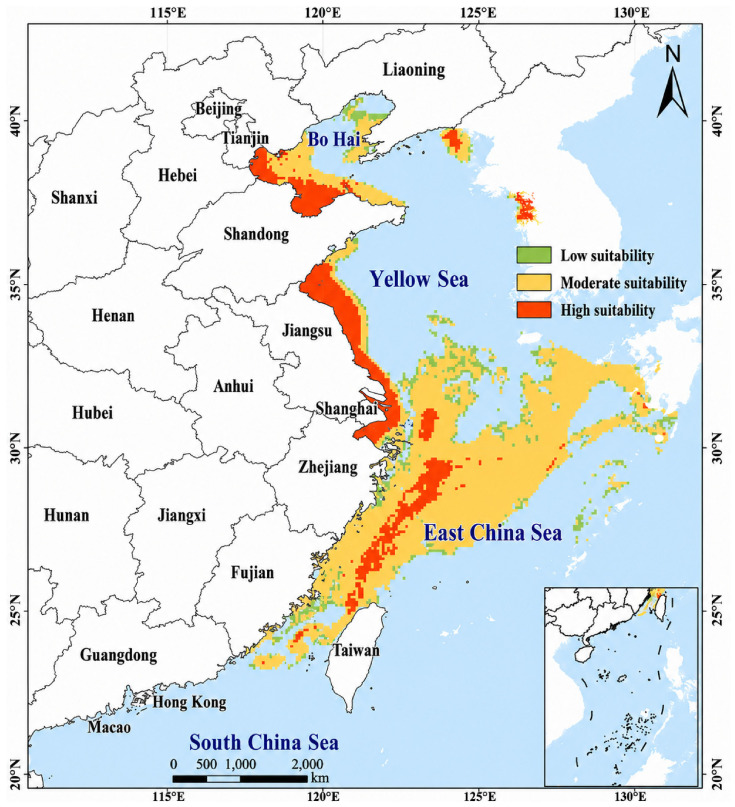
Current potential habitat distribution of *Cryptophyta* in the China Seas. Habitat suitability was classified into low, moderate, and high suitability based on ensemble-model predictions.

Under current climatic conditions, the ensemble model predicted that suitable habitats for Cryptophyta were widely distributed in the China Seas, with a clear nearshore and shelf-oriented pattern (Figure 6). Suitable areas were mainly concentrated along the Bohai Sea, Yellow Sea, East China Sea, and parts of the northern South China Sea. High-suitability habitats occurred primarily in coastal and semi-enclosed waters, where relatively stable salinity, moderate temperature, and weaker water exchange may provide suitable recipient environmental conditions for Cryptophyta occurrence [25,27].

Moderate-suitability habitats formed the dominant distribution class and extended broadly across eastern coastal and continental-shelf waters. These areas were especially prominent from the Yellow Sea to the East China Sea, indicating that Cryptophyta may have a wide ecological tolerance under present marine environmental conditions [28,29]. Low-suitability habitats were distributed mainly around the margins of suitable zones and in waters where hydrodynamic or salinity conditions may be less favorable for long-term establishment.

The total suitable habitat area under present conditions was 63.55 × 10^4^ km^2^, accounting for a substantial proportion of the modeled marine area (Table 3). Among the suitability classes, moderate-suitability habitats occupied the largest area (43.75 × 10^4^ km^2^), followed by high-suitability habitats (13.29 × 10^4^ km^2^) and low-suitability habitats (6.51 × 10^4^ km^2^). The dominance of moderate- and high-suitability areas suggests that current environmental conditions in large parts of China’s coastal seas are predicted to be environmentally suitable for Cryptophyta occurrence, particularly in nearshore and shelf regions influenced by temperature and salinity gradients.

### 3.4. Future Habitat Suitability and Spatial Turnover Under Climate Scenarios

**Figure 7:**
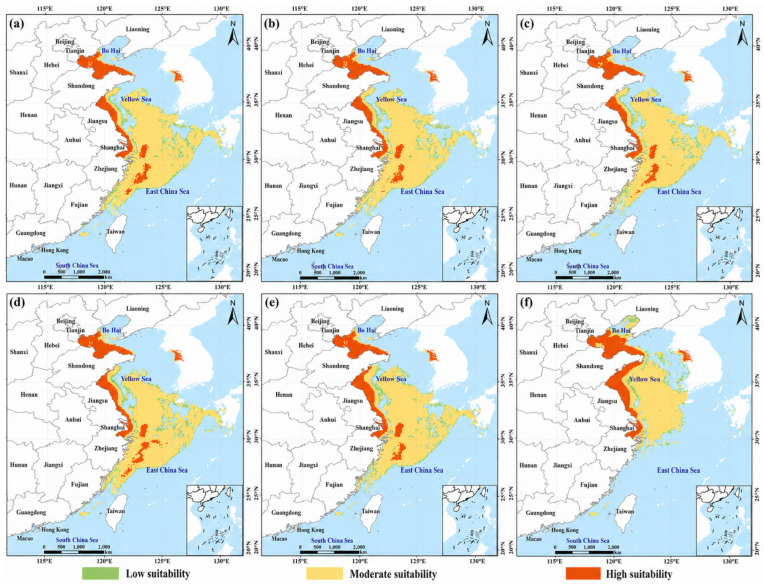
Predicted potential distribution of Cryptophyta under future climate scenarios. (**a**–**c**) represent projections for the 2050s under RCP2.6, RCP4.5, and RCP8.5, respectively; (**d**–**f**) represent projections for the 2100s under RCP2.6, RCP4.5, and RCP8.5, respectively. Habitat suitability was classified into low, moderate, and high suitability based on ensemble-model predictions.

Future projections showed that Cryptophyta suitable habitats would remain extensive under most climate scenarios, but their suitability levels and spatial stability varied markedly with emission intensity and time period (Figure 7; Table 3 and Table 4). Compared with the current suitable habitat area of 63.55 × 10^4^ km^2^, total suitable area increased slightly under RCP2.6 and RCP4.5, whereas a clear reduction occurred under RCP8.5 by the 2100s. This indicates that moderate climate change may maintain a broad potential environmental suitability for Cryptophyta, while strong warming may drive more substantial reorganization of suitable habitat patterns [30].

By the 2050s, total suitable habitat area reached 66.94 × 10^4^ km^2^ under RCP2.6, 63.77 × 10^4^ km^2^ under RCP4.5, and 63.92 × 10^4^ km^2^ under RCP8.5. Moderate-suitability habitats remained the dominant class in all scenarios, ranging from 44.04 to 47.44 × 10^4^ km^2^, while high-suitability habitats changed only slightly (13.25–13.69 × 10^4^ km^2^). These results suggest that mid-century climate change would mainly alter the internal composition of suitability classes rather than causing a large-scale loss of potentially suitable habitats [30].

By the 2100s, the divergence among scenarios became more evident. Under RCP2.6 and RCP4.5, total suitable habitats remained relatively high, reaching 66.71 × 10^4^ km^2^ and 65.86 × 10^4^ km^2^, respectively. Under RCP8.5, however, total suitable area decreased to 54.32 × 10^4^ km^2^. The most notable change was the sharp reduction in moderate-suitability habitats to 32.11 × 10^4^ km^2^, while high-suitability habitats increased to 16.79 × 10^4^ km^2^. This pattern suggests that severe warming may reduce broadly suitable areas while increasing predicted suitability in more limited regions [30,31].

Expansion, contraction, and persistence analyses further clarified the spatial turnover of suitable habitats (Table 4). In the 2050s, expansion was slightly greater than contraction under all three scenarios, with expanded areas ranging from 16.26 to 17.36 × 10^4^ km^2^ and contracted areas ranging from 13.54 to 16.95 × 10^4^ km^2^. The unchanged suitable area remained large (46.50–49.91 × 10^4^ km^2^), indicating that most current suitable habitats would persist through the mid-century period. By the 2100s, spatial turnover became more scenario-dependent. Under RCP2.6, persistence remained high, with 51.42 × 10^4^ km^2^ of unchanged suitable habitat. Under RCP4.5, both expansion (20.70 × 10^4^ km^2^) and contraction (18.36 × 10^4^ km^2^) increased, indicating more active redistribution. The strongest reorganization occurred under RCP8.5, where contraction rose to 38.84 × 10^4^ km^2^ and unchanged suitable area declined to 24.61 × 10^4^ km^2^, despite an expansion of 29.68 × 10^4^ km^2^. Uncertainty in turnover estimates may be higher near the suitability threshold and in areas with weaker agreement among retained model runs, especially under RCP8.5 by the 2100s. Overall, these results suggest that high-emission conditions may reduce the spatial persistence of suitable habitats and intensify habitat redistribution by the 2100s, although the projected turnover should be interpreted as potential suitability change under the selected climate scenarios rather than as a fixed distributional outcome.

### 3.5. Centroid Migration of Suitable Habitats

**Figure 8:**
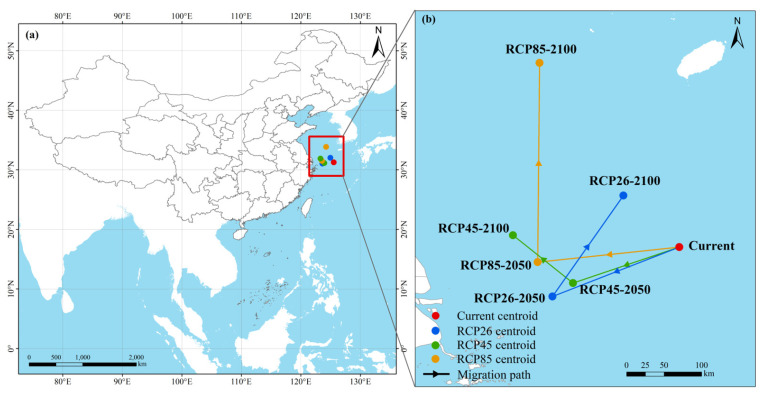
Migration trajectories of the centroid of suitable habitats for Cryptophyta under current and future climate scenarios. (**a**) Geographic location of the study area and centroid positions of suitable habitats under current and future scenarios. (**b**) Enlarged view of centroid migration trajectories under RCP2.6, RCP4.5, and RCP8.5 scenarios in the 2050s and 2100s. Colored dots represent centroid locations for the current period and future scenarios, and arrows indicate the direction of centroid movement.

Centroid migration analysis revealed directional shifts in the spatial center of Cryptophyta suitable habitats under future climate scenarios (Figure 8). Under current conditions, the centroid was located in the East China Sea region, reflecting the broad concentration of suitable habitats across eastern coastal and shelf waters. By the 2050s, centroid displacement was relatively limited under RCP2.6 and RCP4.5, whereas RCP8.5 produced a more evident shift, indicating stronger redistribution of suitable habitats under higher warming pressure.

By the 2100s, the contrast among scenarios became clearer. The centroid under RCP2.6 remained relatively stable, consistent with the high persistence of suitable habitats under the low-emission pathway. Under RCP4.5, the centroid showed moderate displacement, corresponding to increased spatial turnover. Under RCP8.5, the centroid moved more markedly, suggesting that severe warming may drive a directional redistribution of Cryptophyta suitable habitats rather than a simple uniform expansion or contraction. Overall, the centroid trajectories support the area-based results, showing limited shifts under lower-emission scenarios and stronger spatial reorganization under the high-emission pathway.

## 4. Discussion

### 4.1. Reliability of Ensemble Modeling for Cryptophyta Distribution Prediction

The high predictive accuracy of the ensemble model indicates that the Biomod2 framework is suitable for assessing the potential distribution of Cryptophyta in the China Seas. Although several individual algorithms performed well, their stability differed markedly. RF and GBM showed strong predictive ability, reflecting their capacity to capture nonlinear responses to environmental gradients, whereas MaxEnt and SRE were less stable. This difference supports the use of ensemble modeling [14], especially for microalgal taxa whose occurrence records are often spatially uneven and whose environmental responses may be complex [16].

This issue is particularly relevant because Cryptophyta was modeled at the taxonomic-group level rather than as a single species. Different cryptophyte taxa may have distinct physiological tolerances, and group-level records therefore represent a composite ecological signal [32]. Ensemble modeling can reduce the dependence on any single algorithmic assumption and provide a more conservative basis for identifying broad-scale suitable habitats. Nevertheless, the model outputs should be interpreted as potential environmental suitability rather than direct predictions of abundance, bloom intensity, or invasion success. Future species-level analyses, supported by occurrence records and physiological-response data for individual cryptophyte taxa, would help refine the interpretation of taxon-specific climate responses and improve risk assessment.

### 4.2. Temperature and Salinity as Key Constraints on Cryptophyta Habitats

Annual mean temperature and maximum salinity were the two most important predictors of Cryptophyta habitat suitability, suggesting that thermal and osmotic conditions jointly define the large-scale distribution template of this group. Temperature affects photosynthesis, respiration, enzymatic activity, cell division, and seasonal succession of phytoplankton [33]. Therefore, changes in long-term thermal conditions may strongly influence whether Cryptophyta populations can persist in coastal waters.

Salinity was another key constraint, which is consistent with the strong estuarine and coastal gradients in the China Seas [11,18]. River discharge, monsoon circulation, shelf water exchange, and semi-enclosed coastal systems create spatially heterogeneous salinity conditions. Although many Cryptophyta can tolerate brackish to marine environments, their persistence still depends on whether salinity remains within a suitable range [2]. Thus, temperature may determine the broad climatic window for Cryptophyta occurrence, whereas salinity further filters suitable habitats along coastal and shelf gradients.

The lower importance of current velocity, salinity range, and ice thickness does not mean that these factors are ecologically unimportant. Their effects are more likely to operate at regional or local scales. Hydrodynamic conditions can influence dispersal, dilution, and retention of algal cells, especially in ports, estuaries, aquaculture waters, and semi-enclosed bays [27,34]. Ice-related variables mainly constrain northern marginal seas and therefore contribute less to the overall distribution across the China Seas. These results suggest a hierarchical control mechanism in which temperature and salinity shape broad-scale suitability, while hydrodynamic and regional factors regulate local habitat expression.

### 4.3. Climate-Driven Redistribution of Cryptophyta Suitable Habitats

Climate-driven redistribution represents one of the most visible ecological responses to changing environmental conditions. For many taxa, climate change does not immediately cause local extinction or physiological adaptation, but first reshapes where suitable habitats can persist or emerge [35]. Under low- to moderate-emission pathways, suitable habitats remained broadly persistent, indicating that current coastal-shelf environments may continue to support Cryptophyta under moderate hydroclimatic change. By contrast, the high-emission pathway produced a net decline in total suitable area by the 2100s, together with a shift from moderate suitability toward more spatially restricted high-suitability patches. This pattern points to habitat compression: favorable conditions may intensify locally while the broader environmental envelope contracts.

This nonlinear response is consistent with the broader view that phytoplankton distributions are governed by interacting thermal, salinity, stratification, nutrient, and circulation controls [9,36]. Moderate warming can relax environmental constraints in some coastal waters, but stronger warming and salinity reorganization may exceed physiological or ecological optima and reduce broadly suitable habitat [10,36]. Similar climate sensitivity has been reported for HAB taxa and other marine microorganisms, although the direction of redistribution varies with taxon-specific tolerance and regional hydrography [9,10,36]. Therefore, the Cryptophyta response observed here should be interpreted as scenario-dependent restructuring rather than a uniform climate-driven increase in risk.

The centroid and turnover analyses reinforce this interpretation. Limited centroid displacement under lower-emission scenarios implies persistence of an East China Sea coastal-shelf core, whereas stronger displacement and reduced persistence under RCP8.5 indicate spatial replacement under severe forcing. Importantly, these projections describe potential environmental suitability, not realized abundance, bloom occurrence, or bioinvasion success [37]. As with other SDM applications, inference is constrained by occurrence-data structure, model transferability, dispersal processes, and unmeasured biological or anthropogenic drivers [22].

### 4.4. Habitat-Suitability-Based Surveillance Priorities in Coastal Waters

**Figure 9:**
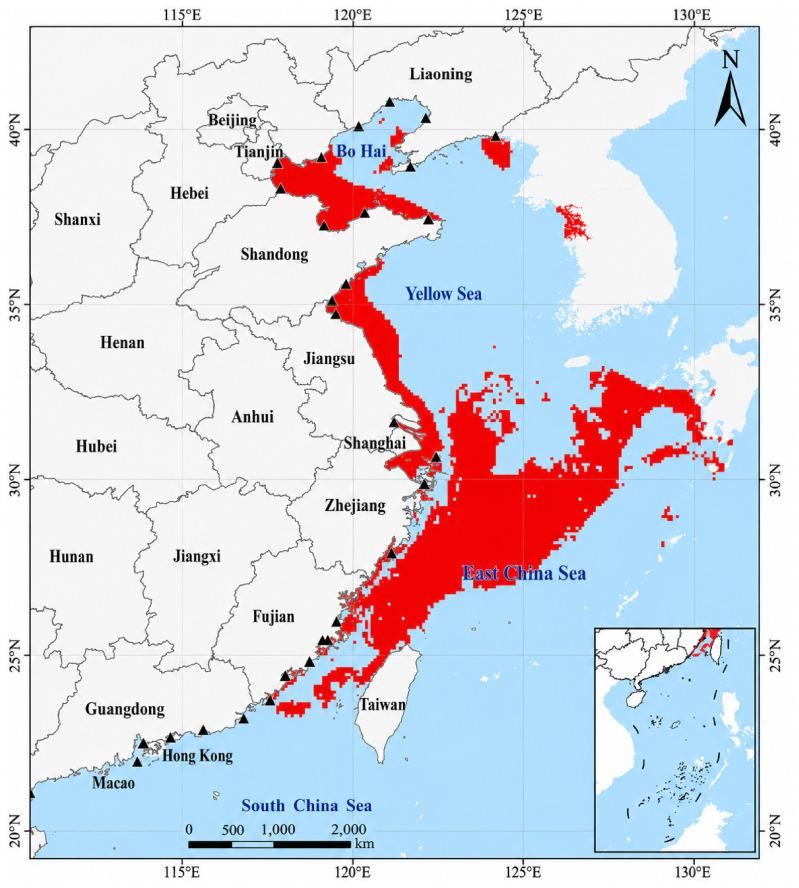
Habitat-suitability-based surveillance priority zones for Cryptophyta in the China Seas based on ensemble model predictions. The highlighted regions represent areas with high or persistent habitat suitability under current and future climate scenarios.

The projected surveillance-priority zones were identified primarily from persistent or high habitat suitability patterns and should therefore be interpreted as habitat-suitability-based screening areas (Figure 9). Rather than being uniformly distributed across the China Seas, areas of persistent or high suitability were concentrated in the Bohai Sea, western Yellow Sea coast, and East China Sea shelf, particularly from the Yangtze River estuary to the Zhejiang-Fujian coastal waters. This spatial pattern is broadly consistent with distributional assessments of other harmful or ballast-water-associated microalgae in Chinese coastal waters, such as *Alexandrium* spp., for which Bohai Bay, the Yangtze River estuary–Fujian coastal belt, and parts of the southern China coast have also been identified as suitable or surveillance-priority regions [38]. These regions combine suitable thermal-salinity conditions with dense port networks, estuarine exchange, aquaculture activity, and semi-enclosed or shelf systems that may favor retention [11,18]. The overlap among different microalgal groups suggests that these coastal areas may represent recurrent monitoring-priority zones, although the relative importance of each region can vary among taxa because of differences in physiological tolerance, life-history traits, and dominant environmental drivers.

The East China Sea appears especially important because it forms a broad and relatively continuous suitability core. Its coastal-shelf gradients, riverine influence, and water-mass exchange can generate heterogeneous but persistent niches for opportunistic phytoplankton [16]. The Bohai Sea represents a different type of concern: weak water exchange, intensive coastal use, and eutrophication-sensitive conditions may enhance local accumulation once viable propagules are introduced [5,6]. Thus, priority areas reflect different mechanisms of concern, from broad habitat persistence on the shelf to local retention in semi-enclosed waters.

Nevertheless, these zones should be interpreted as habitat-suitability-based surveillance priorities rather than confirmed ecological-risk, invasion, or bloom hotspots. Ballast-water-mediated risk requires the overlap of suitable recipient environments with propagule supply, port connectivity, organism viability, and post-discharge establishment conditions [7]. The model, therefore, provides a spatial screening layer that can guide monitoring design, but it should be combined with ballast-water discharge records, molecular detection, nutrients, hydrodynamic retention, and local ecological observations before drawing strong risk conclusions [7,39].

### 4.5. Implications for Monitoring, Management, and Future Perspectives

These findings support a transition from static coastal monitoring toward climate-informed surveillance of ballast-water-associated microalgae. Temperature and salinity can serve as practical early-warning indicators because they were the dominant predictors of Cryptophyta suitability, but they should not be used in isolation. A more robust monitoring framework would combine suitability maps with port activity, ballast-water discharge, aquaculture distribution, hydrodynamic retention, and molecular identification of cryptophyte taxa [7].

From a management perspective, monitoring effort should prioritize persistent or newly suitable habitats and be further refined using independent information on port activity, shipping intensity, ballast-water discharge, aquaculture distribution, and hydrodynamic retention. The Bohai Sea, Yangtze River estuary, Zhejiang-Fujian coast, Taiwan Strait, and adjacent East China Sea shelf waters are therefore suitable candidates for long-term surveillance. In these areas, routine microscopy should be complemented by molecular tools, particularly because cryptophytes are small, morphologically similar, and often resolved only at broad taxonomic levels in conventional monitoring.

Several uncertainties should guide future work. First, group-level modeling may obscure species-specific differences in ecological tolerance, introduction status, and introduction and establishment potential. Second, the present analysis focused mainly on physical environmental predictors and did not explicitly include nutrients, grazing pressure, species interactions, resting stages, or direct propagule pressure. Third, future projections assume that present-day environment-occurrence relationships remain transferable under changing climate conditions. Future studies should integrate species-level records, ballast-water source data, environmental DNA-based metabarcoding or targeted molecular assays for detecting cryptophyte taxa, and process-based coastal observations to move from habitat suitability toward more mechanistic risk assessment.

## 5. Conclusions

This study applied an ensemble species distribution modeling framework to assess the current and future habitat suitability of Cryptophyta across the Bohai Sea, Yellow Sea, East China Sea, and South China Sea under climate-change scenarios. The results indicate that Cryptophyta habitat suitability is mainly constrained by annual mean temperature and maximum salinity, and its future response is scenario-dependent rather than a simple linear expansion. Under low- to moderate-emission scenarios, suitable habitats are projected to remain broadly distributed or increase slightly, whereas high-emission forcing by the 2100s may reduce suitable areas and promote stronger spatial reorganization. These changes suggest that climate-driven habitat redistribution may alter the spatial pattern of potential Cryptophyta occurrence in China’s coastal waters. From a management perspective, suitable habitats should be interpreted as potential recipient environments rather than direct evidence of invasion or bloom occurrence. Because ballast water can transport viable microalgae across regions, priority surveillance should focus on areas where persistent or newly suitable habitats overlap with intensive port activities, estuaries, semi-enclosed bays, aquaculture zones, and major shipping routes. In particular, the Bohai Sea, the Yellow Sea coasts, the East China Sea suitability cores, and emerging suitable coastal zones in the South China Sea deserve continued attention. These findings provide a spatial basis for climate-informed monitoring, early warning, and adaptive surveillance and management of ballast-water-associated microalgae in the China Seas.

## Figures and Tables

**Table 1:** Environmental variables involved in modeling (24 predictors).

Abbreviation	Environment Variable	Unit
bio1	Maximum monthly mean current velocity	m/s
bio2	Minimum monthly mean current velocity	m/s
bio3	Maximum current velocity	m/s
bio4	Annual mean current velocity	m/s
bio5	Minimum current velocity	m/s
bio6	Annual current velocity range	m/s
bio7	Maximum monthly mean ice thickness	m
bio8	Minimum monthly mean ice thickness	m
bio9	Maximum ice thickness	m
bio10	Annual mean ice thickness	m
bio11	Minimum ice thickness	m
bio12	Annual ice thickness range	m
bio13	Maximum monthly mean salinity	PSU
bio14	Minimum monthly mean salinity	PSU
bio15	Maximum salinity	PSU
bio16	Annual mean salinity	PSU
bio17	Minimum salinity	PSU
bio18	Annual salinity range	PSU
bio19	Maximum monthly mean temperature	°C
bio20	Minimum monthly mean temperature	°C
bio21	Maximum temperature	°C
bio22	Annual mean temperature	°C
bio23	Minimum temperature	°C
bio24	Annual temperature range	°C

**Table 2:** Accuracy evaluation of individual distribution models based on Kappa, TSS, and AUC values.

Model	KAPPA_Mean	KAPPA_sd	AUC_Mean	AUC_sd	TSS_Mean	TSS_sd
ANN	0.8149	0.1001	0.9339	0.0376	0.8193	0.0985
CTA	0.8007	0.1130	0.9312	0.0427	0.8034	0.1118
GAM	0.8279	0.0628	0.9379	0.0278	0.8302	0.0625
GBM	0.8624	0.0795	0.9728	0.0222	0.8646	0.0788
GLM	0.7293	0.0778	0.8848	0.0391	0.7348	0.0762
MARS	0.8177	0.0653	0.9380	0.0392	0.8206	0.0654
MaxEnt	0.6116	0.1614	0.8082	0.0927	0.6110	0.1636
RF	0.8658	0.0617	0.9781	0.0173	0.8671	0.0613
SRE	0.5953	0.0674	0.7968	0.0338	0.5938	0.0676

_sd represents the standard deviation calculated from 10 replicate runs.

**Table 3:** Predicted area of suitable habitats for Cryptophyta under current and future climate scenarios (10^4^ km^2^).

Period	Low-Suitability Area	Moderate-Suitability Area	High-Suitability Area
present	6.51	43.75	13.29
2050_RCP2.6	5.87	47.44	13.64
2050_RCP4.5	6.34	44.19	13.25
2050_RCP8.5	6.19	44.04	13.69
2100_RCP2.6	6.46	45.64	14.61
2100_RCP4.5	6.23	44.27	15.36
2100_RCP8.5	5.42	32.11	16.79

**Table 4:** Expansion, contraction, and persistence of Cryptophyta suitable habitats under future climate scenarios (10^4^ km^2^).

Period	Contraction	Expansion	Unchanged
2050_RCP2.6	13.54	16.96	49.91
2050_RCP4.5	16.04	16.26	47.41
2050_RCP8.5	16.95	17.36	46.50
2100_RCP2.6	12.03	15.21	51.42
2100_RCP4.5	18.36	20.70	45.09
2100_RCP8.5	38.84	29.68	24.61

## Data Availability

The datasets generated and analyzed during the current study are available from the corresponding author on reasonable request.

## Supplementary Materials

The following supporting information can be downloaded at: https://www.mdpi.com/article/10.3390/biology15131047/s1, The supporting information includes Texts S1 and S2, Figure S1 and Tables S1–S5, providing details on occurrence-record processing, pseudo-absence/background generation, representative microscopic observation, port-related background information, occurrence coordinates, environmental-data sources, and variable-screening results.
