# Supplementary material for: Assessing Climate Change Impacts on the Distribution and Ecological Risks of Cryptophyta in the China Seas Using Ensemble Models
Source: Biology (Basel). 2026 Jul 1;15(13):1047. doi: 10.3390/biology15131047 (PMC13359735; doi:10.3390/biology15131047)
Supplement: Supplementary file 1 [file biology-15-01047-s001.zip › biology-4379101-supplementary.pdf]

Supporting materials

Assessing Climate Change Impacts on the Distribution and Ecological Risks of Cryptophyta in the China Seas Using Ensemble Models

Ru Lan [1,2], Jing Li [3,4], Rongchang Chen [1], Zhentian Cai [5], Luning Li [1,*]

1 Environmental Protection & Energy-Saving Technique Research Center, China Waterborne Transport Research Institute, Beijing 100088, China;
lanru@wti.ac.cn (R.L.); chenrongchang@wti.ac.cn (R.C.)

2 School of Energy and Environmental Engineering, University of Science and Technology Beijing, Beijing 100083, China

3 Ocean and Food School, Quanzhou Normal University, Quanzhou 362000, China; leekin@qztc.edu.cn

4 Key Laboratory of Coastal Resource Biotechnology, Fujian Higher Institution, Quanzhou 362000, China

5 Jinjiang College, Sichuan University, Meishan 620860, China; 18990186869@163.com

* Correspondence: liluning@wti.ac.cn

Text S1. Sources and processing of *Cryptophyta* occurrence records

Cryptophyta occurrence records were extracted from a database of suspected alien microalgae in Chinese coastal port waters. The database integrated coastal phytoplankton monitoring records, port-water survey records, ballast water samples, and ballast-tank sediment samples from inbound vessels. Records assigned to *Cryptophyta* were retained only when taxonomic information and geographic coordinates were available. Because most monitoring records identified cryptophytes at the group level, *Cryptophyta* was used as the modeling unit rather than a single species.

The broader database was compiled from environmental impact assessment reports of major coastal port projects, marine environmental bulletins, meteorological forecast information, field surveys conducted during 2017–2022, and environmental impact assessment reports collected by the China Waterborne Transport Research Institute during 2012–2022. Environmental-status and marine water-quality data covered 95 engineering/project records from 42 ports in 11 coastal provinces or municipalities, with a temporal range of 2002–2016. These materials provided the background information for coastal port conditions, ballast-water activities, and potential sampling locations. Only records with valid taxonomic assignment and geographic coordinates were retained as occurrence records for model construction.

Ballast-water-related records were supported by samples from 51 inbound vessels and surface ballast-tank sediment samples from 22 inbound vessels. Ballast water samples were examined using microscopic observation and 18S rDNA-based molecular identification. For ballast-tank sediment, surface sediment samples were stored at 4 °C, homogenized, diluted with sterile filtered seawater, ultrasonically dispersed for 2 min, and sequentially filtered through 125 μm and 20 μm sieves. The concentrated suspension was then used for microscopic observation, cell counting, algal isolation, and molecular identification. Representative cryptophyte-like cells were isolated and cultured in f/2 medium for taxonomic confirmation. The 18S rDNA sequences were compared with reference sequences in the NCBI database, and phylogenetic confirmation was performed using maximum-likelihood and neighbor-joining methods

in MEGA.

Before modeling, all records were screened for taxonomic consistency, coordinate accuracy, and spatial validity. Records with missing coordinates, duplicated locations, ambiguous locality information, or positions outside the marine study area were removed. After screening, 136 georeferenced *Cryptophyta* occurrence records were retained. All records were standardized to the WGS84 coordinate system, imported into Microsoft Excel, saved in CSV format, and matched with environmental raster layers before ensemble species distribution modeling..

Text S2. Pseudo-absence/background point generation

Because the *Cryptophyta* occurrence dataset consisted of presence-only records, pseudo-absence/background points were generated to provide contrast data for algorithms requiring absence or background information in the Biomod2 framework. A total of 1000 pseudo-absence/background points were randomly generated across the accessible marine study area, including the Bohai Sea, Yellow Sea, East China Sea, and South China Sea. Raster cells containing valid *Cryptophyta* occurrence records were excluded from pseudo-absence/background sampling where applicable. Based on the 136 valid *Cryptophyta* occurrence records, the pseudo-absence/background-to-presence ratio was approximately 7.35:1.

These pseudo-absence/background points were used only as contrast/background data and should not be interpreted as confirmed absence records. The modeling dataset was divided into training and testing subsets using a 75%/25% split, with 75% of the data used for model calibration and 25% used for model evaluation. To reduce uncertainty associated with random data partitioning and model fitting, each algorithm was run 10 times under the same modeling framework.

No independent field-verified absence records were available. Therefore, no confirmed-absence-based correction was applied. To reduce potential errors in occurrence data, all records were quality-controlled before modeling by removing duplicated records, records with missing or ambiguous coordinates, and records outside the marine study area.

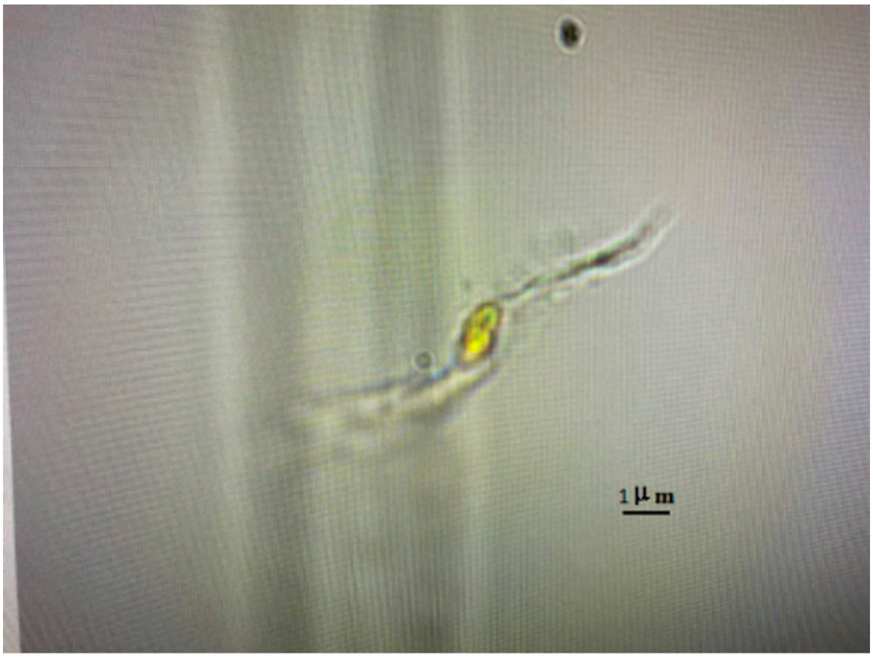

Figure S1. Representative cryptophyte-like algal cell observed under light microscopy. Scale bar = 1 µm.

Table S1. Summary of environmental-status and marine water-quality data used to support occurrence-record screening

Data category	Specific information	Data sources	Temporal range
Environmental status	Climate conditions; air temperature; precipitation; wind conditions; sampling time; sampling station	Environmental impact assessment reports of major coastal port projects; marine environmental bulletins; meteorological forecast information	2002–2016
Marine water quality (monitoring values)	Water temperature; salinity; inorganic nitrogen; active phosphate; dissolved oxygen; petroleum hydrocarbons	Environmental impact assessment reports of major coastal port projects; marine environmental bulletins; meteorological forecast information	2002–2016

Table S2. Coastal provinces/municipalities and ports included in the compiled port-related database.

Province/municipality	Ports included
Liaoning	Jinzhou Port; Suizhong Port; Dalian Port; Dandong Port; Yingkou Port; Huludao Port
Hebei	Tangshan Caofeidian Port Area; Tangshan/Jingtang Port Area; Huanghua Port
Tianjin	Tianjin Port
Shandong	Penglai Port; Yantai Port; Binzhou Port; Qingdao Port; Rizhao Port; Weifang Port
Jiangsu	Nantong Port; Taizhou Port; Suzhou Port; Zhenjiang Port; Lianyungang Port
Zhejiang	Zhoushan Port; Wenzhou Port; Ningbo Port; Yangshan Deep-water Port; Zhapu Port
Shanghai	Shanghai Port
Fujian	Quanzhou Port; Fuzhou Port; Xiamen Port; Putian Xinghua Port
Guangdong	Guangzhou Port; Shenzhen Port; Zhanjiang Port; Jieyang Port; Shantou Port; Yunfu Port
Guangxi	Qinzhou Port; Beibu Gulf Port
Hainan	Haikou Port; Yangpu Port; Wenchang Puqian Fishing Port

Table S3. Georeferenced occurrence records of Cryptophyta used for ensemble species distribution modeling

Record ID	Longitude (°E)	Latitude (°N)	Record ID	Longitude (°E)	Latitude (°N)
1	124.154	39.786	69	120.270	34.928
2	124.036	39.776	70	120.370	34.028
3	124.141	39.768	71	119.512	34.715
4	124.113	39.759	72	119.674	34.805
5	124.151	39.751	73	119.933	34.911
6	123.984	39.751	74	119.605	34.643
7	124.023	39.743	75	119.692	34.743
8	124.061	39.734	76	119.723	34.746
9	124.099	39.726	77	119.865	34.837
10	124.138	39.718	78	120.005	35.014
11	123.970	39.717	79	120.683	32.007
12	124.009	39.709	80	120.672	32.005
13	124.047	39.701	81	120.693	32.009
14	124.085	39.692	82	120.666	32.005
15	124.124	39.684	83	120.596	32.009
16	123.957	39.684	84	120.586	32.036
17	123.995	39.675	85	120.646	32.062
18	124.033	39.667	86	120.672	32.058
19	124.072	39.658	87	120.686	32.052
20	124.110	39.650	88	120.734	32.035
21	117.958	38.376	89	119.236	32.213
22	117.958	38.357	90	119.253	32.203
23	117.892	38.336	91	119.276	32.192
24	118.914	38.329	92	119.222	32.218
25	117.900	38.318	93	119.232	32.214
26	117.923	38.348	94	113.594	23.100
27	117.896	38.333	95	113.599	23.106
28	117.881	38.321	96	113.606	23.108
29	119.873	32.266	97	112.021	23.082
30	119.864	32.280	98	112.028	23.077
31	119.855	32.288	99	112.028	23.039
32	119.852	32.290	100	124.500	31.000
33	119.848	32.293	101	124.000	31.000
34	119.421	34.795	102	123.000	31.000
35	119.514	34.852	103	129.100	29.100
36	119.608	34.917	104	122.800	28.000
37	119.718	34.992	105	120.983	27.833
38	119.831	35.062	106	121.400	26.400
39	119.955	35.144	107	121.755	26.230
40	120.084	35.236	108	122.060	26.050
41	119.487	34.742	109	123.500	31.000

Record ID	Longitude (°E)	Latitude (°N)	Record ID	Longitude (°E)	Latitude (°N)
42	119.575	34.801	110	121.000	27.833
43	119.690	34.870	111	123.220	27.790
44	119.792	34.935	112	124.200	28.800
45	119.916	35.010	113	127.700	23.200
46	120.031	35.094	114	121.450	26.410
47	120.154	35.186	115	121.500	26.400
48	119.533	34.662	116	124.070	26.470
49	119.640	34.733	117	121.800	26.200
50	119.767	34.812	118	121.500	27.200
51	119.857	34.878	119	122.500	32.000
52	119.999	34.946	120	123.500	32.000
53	120.231	35.132	121	124.500	32.000
54	119.629	34.604	122	125.000	30.000
55	119.834	34.761	123	121.500	27.100
56	119.925	34.833	124	122.820	28.040
57	119.732	34.564	125	124.630	28.640
58	119.812	34.627	126	123.100	29.500
59	119.902	34.711	127	123.400	29.300
60	119.992	34.793	128	123.800	29.100
61	120.093	34.885	129	122.800	28.100
62	120.198	34.980	130	122.170	28.450
63	120.301	34.079	131	123.800	27.200
64	119.843	34.503	132	123.700	29.100
65	119.911	34.575	133	123.200	27.700
66	119.993	34.650	134	121.900	27.800
67	120.078	34.731	135	121.500	27.180
68	120.177	34.831	136	121.700	26.200

Table S4. Environmental data sources and preprocessing information for the six retained predictors used in the final ensemble model.

Predictor	Environmental variable	Unit	Layer setting	Current data source	Processed resolution	Temporal baseline	Future scenario source
bio2	Minimum monthly mean current velocity	m s^{-1}	Surface marine layer	Bio-ORACLE v2.1	5 arcmin	Present-day climatology provided by Bio-ORACLE v2.1	Processed Bio-ORACLE climate products under RCP2.6, RCP4.5, and RCP8.5 for the 2050s and 2100s
bio3	Maximum current velocity	m s^{-1}	Surface marine layer	Bio-ORACLE v2.1	5 arcmin	Present-day climatology provided by Bio-ORACLE v2.1	Processed Bio-ORACLE climate products under RCP2.6, RCP4.5, and RCP8.5 for the 2050s and 2100s
bio9	Maximum ice thickness	m	Surface marine layer	Bio-ORACLE v2.1 [19]	5 arcmin	Present-day climatology provided by Bio-ORACLE v2.1	Processed Bio-ORACLE climate products under RCP2.6, RCP4.5, and RCP8.5 for the 2050s and 2100s
bio15	Maximum salinity	PSU	Surface marine layer	Bio-ORACLE v2.1 and WOA2018	5 arcmin	Present-day climatology harmonized from Bio-ORACLE v2.1 and WOA2018	Processed Bio-ORACLE climate products under RCP2.6, RCP4.5, and RCP8.5 for the 2050s and 2100s
bio18	Annual salinity range	PSU	Surface marine layer	Bio-ORACLE v2.1 and WOA2018	5 arcmin	Present-day climatology harmonized from Bio-ORACLE v2.1 and WOA2018	Processed Bio-ORACLE climate products under RCP2.6, RCP4.5, and RCP8.5 for the 2050s and 2100s
bio22	Annual mean temperature	°C	Surface marine layer	Bio-ORACLE v2.1 and WOA2018	5 arcmin	Present-day climatology harmonized from Bio-ORACLE v2.1 and	Processed Bio-ORACLE climate products under RCP2.6, RCP4.5, and

	WOA2018	RCP8.5 for the 2050s and 2100s

Note: All layers were clipped to the China Seas marine study area, masked to marine cells, standardized to WGS84, resampled to a common 5 arcmin grid, and aligned to the same spatial extent before environmental values were extracted. Future layers were obtained from processed Bio-ORACLE climate products; no additional GCM selection or downscaling was performed in this study.

Table S5. Summary of excluded environmental variables and reasons for retaining final predictors after Pearson correlation screening

Excluded predictor	Retained counterpart	Pearson's r	Reason for exclusion or retention
bio1	bio3, maximum current velocity	0.988	Highly correlated with bio3. bio3 was retained to represent the upper hydrodynamic constraint and dispersal potential.
bio4	bio3, maximum current velocity	0.980	Highly correlated with bio3. bio3 was retained because maximum velocity is a more direct hydrodynamic constraint.
bio5	bio2, minimum monthly mean current velocity; bio3, maximum current velocity	0.429; 0.622	Not excluded solely due to high correlation. Absolute minimum current velocity had weaker interpretability and may reflect near-zero or unstable values; bio2 and bio3 represented low-flow and maximum-current conditions more clearly.
bio6	bio3, maximum current velocity	0.993	Highly correlated with bio3, indicating that current range was largely driven by maximum current velocity. bio3 was retained for clearer interpretation.
bio7	bio9, maximum ice thickness	1.000	Almost perfectly correlated with bio9. bio9 was retained to represent the strongest ice-related constraint.
bio8	bio9, maximum ice thickness	NA	Produced undefined correlations, indicating no effective spatial variation within the study area. bio9 provided a clearer ice-related predictor.
bio10	bio9, maximum ice thickness	0.999	Highly correlated with bio9. bio9 was retained because maximum ice thickness better reflects extreme ice-cover constraints.
bio11	bio9, maximum ice thickness	NA	Produced undefined correlations, indicating no effective spatial variation within the study area. bio9 was retained as the representative ice-related predictor.
bio12	bio9, maximum ice thickness	1.000	Perfectly correlated with bio9, suggesting that ice-thickness range was mainly determined by maximum ice thickness. bio9 was retained.
bio13	bio15, maximum salinity	0.988	Highly correlated with bio15. bio15 was retained to represent the upper salinity constraint.
bio14	bio15, maximum salinity; bio18, annual salinity range	0.938; -0.844	Strongly correlated with retained salinity predictors. bio15 and bio18 were retained to represent upper salinity limitation and coastal salinity variability.

Excluded predictor	Retained counterpart	Pearson's r	Reason for exclusion or retention
bio16	bio15, maximum salinity	0.971	Highly correlated with bio15. bio15 was retained as a clearer constraint-related salinity metric.
bio17	bio15, maximum salinity; bio18, annual salinity range	0.901; -0.908	Strongly correlated with both retained salinity predictors. bio18 captured salinity variability, whereas bio15 represented upper salinity limitation.
bio19	bio22, annual mean temperature	0.945	Highly correlated with bio22. bio22 was retained to represent the long-term thermal background.
bio20	bio22, annual mean temperature	0.995	Highly correlated with bio22. bio22 was retained as a stable indicator of overall thermal conditions.
bio21	bio22, annual mean temperature	0.897	Highly correlated with bio22. bio22 was retained to integrate long-term thermal conditions and avoid overemphasizing short-term extremes.
bio23	bio22, annual mean temperature	0.995	Highly correlated with bio22. bio22 was retained to summarize the broad thermal gradient relevant to group-level suitability.
bio24	bio22, annual mean temperature	-0.975	Strongly negatively correlated with bio22. bio22 was retained as a clearer and more directly interpretable thermal predictor.

Note: Variables were considered highly correlated when $|r| > 0.8$. For highly correlated variable groups, one predictor was retained based on ecological relevance to Cryptophyta habitat suitability and environmental interpretability. Variables that did not provide effective spatial variation or showed weaker ecological interpretability were not retained when the same environmental dimension was already represented by more stable predictors. NA indicates that the correlation coefficient could not be calculated because the variable showed no effective variation within the study area. The six retained predictors were bio2, bio3, bio9, bio15, bio18, and bio22. No pairwise correlation among the six retained predictors exceeded the $|r| > 0.8$ threshold.
